# Comparison of common perioperative blood loss estimation techniques: a systematic review and meta-analysis

**DOI:** 10.1007/s10877-020-00579-8

**Published:** 2020-08-19

**Authors:** Lara Gerdessen, Patrick Meybohm, Suma Choorapoikayil, Eva Herrmann, Isabel Taeuber, Vanessa Neef, Florian J. Raimann, Kai Zacharowski, Florian Piekarski

**Affiliations:** 1 Department of Anaesthesiology, Intensive Care Medicine and Pain Therapy, University Hospital Frankfurt, Goethe University Frankfurt, Theodor-Stern-Kai 7, 60590 Frankfurt am Main, Germany; 2 Department of Anaesthesia and Critical Care, University Hospital Würzburg, Würzburg, Germany; 3 Institute of Biostatistics and Mathematical Modelling, Department of Medicine, Goethe University, Frankfurt, Germany

**Keywords:** Blood loss estimation, Visual estimation, Gravimetric method, Patient blood management, Direct measurement, Colorimetric blood loss estimation

## Abstract

Estimating intraoperative blood loss is one of the daily challenges for clinicians. Despite the knowledge of the inaccuracy of visual estimation by anaesthetists and surgeons, this is still the mainstay to estimate surgical blood loss. This review aims at highlighting the strengths and weaknesses of currently used measurement methods. A systematic review of studies on estimation of blood loss was carried out. Studies were included investigating the accuracy of techniques for quantifying blood loss in vivo and in vitro. We excluded nonhuman trials and studies using only monitoring parameters to estimate blood loss. A meta-analysis was performed to evaluate systematic measurement errors of the different methods. Only studies that were compared with a validated reference e.g. Haemoglobin extraction assay were included. 90 studies met the inclusion criteria for systematic review and were analyzed. Six studies were included in the meta-analysis, as only these were conducted with a validated reference. The mixed effect meta-analysis showed the highest correlation to the reference for colorimetric methods (0.93 95% CI 0.91–0.96), followed by gravimetric (0.77 95% CI 0.61–0.93) and finally visual methods (0.61 95% CI 0.40–0.82). The bias for estimated blood loss (ml) was lowest for colorimetric methods (57.59 95% CI 23.88–91.3) compared to the reference, followed by gravimetric (326.36 95% CI 201.65–450.86) and visual methods (456.51 95% CI 395.19–517.83). Of the many studies included, only a few were compared with a validated reference. The majority of the studies chose known imprecise procedures as the method of comparison. Colorimetric methods offer the highest degree of accuracy in blood loss estimation. Systems that use colorimetric techniques have a significant advantage in the real-time assessment of blood loss.

**Electronic supplementary material:**

The online version of this article (10.1007/s10877-020-00579-8) contains supplementary material, which is available to authorized users.

## Introduction

Estimating intraoperative blood loss is a daily challenge of clinicians and until now a standardized method is not being used routinely. Despite the knowledge of the inaccuracy of visual estimation, intraoperative blood loss is still recorded visually [1, 2]. However, the quantified blood loss plays a key role in blood transfusion decisions, along with other information such as hemoglobin values and individual transfusion triggers. Inappropriate transfusion of blood products is associated with risks and influences patient´s outcome [3, 4]. Changes in vital and laboratory parameters recorded during routine monitoring only occur in the event of imminent or manifest hemodynamic instability and organ perfusion diminution.

Especially in obstetrics, estimating blood loss is challenging, as amniotic fluid erroneously causes the impression of high blood loss. In addition, the volume of external blood loss is also difficult to estimate [5, 6]. It is noteworthy that loss of lower blood volumes is estimated more correctly than loss of higher blood volumes. However, large blood loss is life-threatening and therefore more relevant in transfusion decisions [7–9]. Other methods for recording intraoperative blood loss such as pictograms or direct measurement, are rarely used [1]. As part of various programs, the weighing of contaminated material, the so-called gravimetric method, was introduced as a supplement [2, 10–13]. However, the direct measurement by using calibrated collection bags is not used in the operating theatre, whereas this method has long been practiced during childbirth [14].

Within the framework of Patient Blood Management (PBM) as a multidisciplinary and evidence-based treatment concept [4], the recording of blood loss is becoming increasingly important. PBM is based on three main pillars: (1) Anaemia management, (2) Minimizing blood loss and increased use of donor blood saving strategies, (3) Rational use of blood reserves [15]. Reducing blood loss and optimizing patient care starts with the measurement of intraoperative blood loss.

Recently developed methods, such as photometric analysis, are becoming more popular in clinical practice. Three systematic reviews [2, 14, 16] have been published on blood loss estimation in obstetrics. Since then, new approaches and methods have been developed, however, none of these reviews addressed the setting of the operation theatre. This review and meta-analysis are intended to show a range of measurement methods and to highlight the strengths and weaknesses of the methods used within surgery.

## Methods

The protocol of this systematic review was registered in PROSPERO 2020 CRD42020166803 (https://www.crd.york.ac.uk/PROSPERO).

### Data sources and search strategy

The research was carried out using PubMed and Google Scholar databases. Only studies published between 01.01.2000–11.11.2019 in English or German language were considered. This period has been chosen to include primarily current literature with more recent reference methods. The results were merged using the reference management software Citavi 6 (Version 6.3.0.0, Swiss Academic Software GmbH, Wädenswil, Schweiz). For the advanced search on PubMed, synonyms of the word blood loss (blood loss, haemoglobin loss, fluid management) were combined with synonyms of the word measurement (estimate*, measurement, evaluation, determinat*, quantification, quantify, assessment, monitoring). With the feature “Show similar articles” the search has been extended. For the Google Scholar Search, the above stated method refining the PubMed search was also used. Two independent reviewers (LG and FP) screened all abstracts for inclusion and exclusion criteria.

Studies were included investigating the accuracy of techniques for quantifying blood loss in vivo and in vitro. We excluded nonhuman trials and studies using only monitoring parameters to estimate blood loss. Finally, we excluded comments or letters to the editor. Full texts of all selected studies were read and analyzed.

### Assessment of bias

Two reviewers (LG and FP) independently assessed the quality of the included primary studies using the Acrobat-NRSI tool published by the Cochrane Collaboration (https://www.cochrane.de/de/rob-manual). This quality assessment tool evaluates non-randomized studies for bias due to confounders (1), by selecting participants into study groups, (2), by recording the intervention of the performance (3), by deviations in the intervention phase (4), by missing data (5), by endpoint survey (6) and by selective reporting of endpoints (7). After answering the predefined questions for each of the domains, the reviewers have assessed the bias potential for each domain according to the following classification: low Risk of Bias (RoB) “++” (study is comparable to a randomized study); moderate RoB “+” (study correctly performed represents a non-randomized study); significant RoB “−” (study has some serious problems); critical RoB “−−” (study has too many serious problems); unclear RoB “?” (due to lack of information, no assessment is possible). The overall rating of a non-randomized study is based on the domain with the highest RoB.

### Meta-analytical procedures

A meta-analysis was performed to evaluate systematic measurement errors of the different methods. All studies were screened using reference method “hemoglobin extraction assay”. Included in the meta-analysis were studies that used this valid method as reference. The hemoglobin extraction assay is a laborious and therefore an accurate method for quantifying blood volume. In this procedure, blood-soaked products are rinsed and examined to determine the hemoglobin concentration spectrophotometrically [17]. Studies with incomplete data concerning a Bland–Altman analysis were secondarily excluded.

### Statistical analysis

Statistical analyses were carried out with the metafor Package: A Meta-Analysis Package for R (version 2.4-0, Free Software Foundation Inc., Boston, USA) [18]. For the individual studies, the systematic bias ± standard deviation (SD) was extracted from the given Bland Altman analyses. Confidence intervals of bias in the individual studies were calculated. The total bias estimated value with total confidence interval was calculated. The upper and lower tolerance limits were determined. I^2^ statistics were computed to quantify the heterogeneity. If significant heterogeneity of results was found (defined as I^2^ > 50%), we investigated the relationship between this result and the predefined variables outlined above using meta-regression analysis.

Given the heterogeneity of the studies examined in the review, the approach of modelling random effects was used to merge the result data.

## Results

### Study identification

**Fig. 1:**
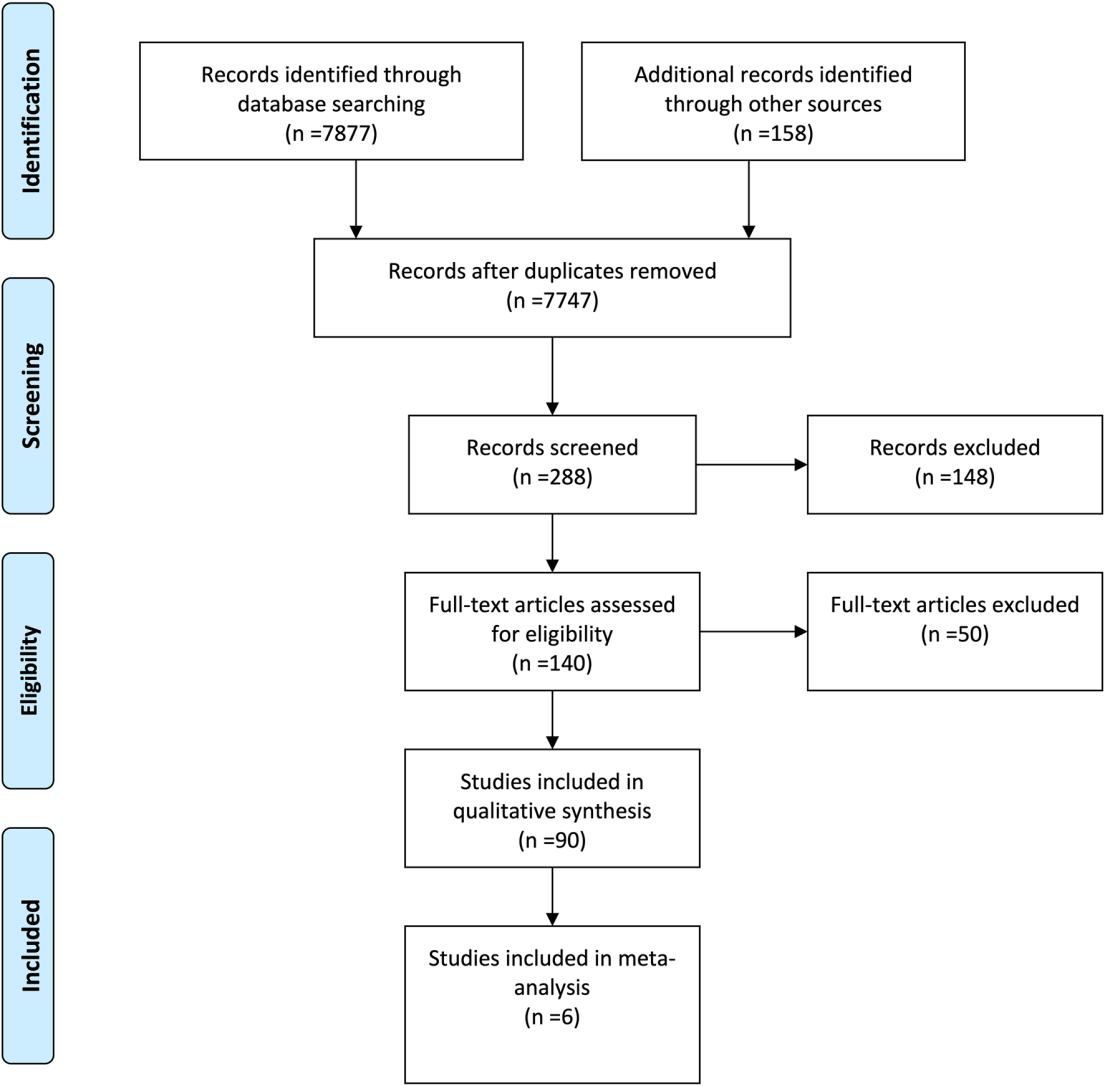
PRISMA flow diagram. From: Moher D, Liberati A, Tetzlaff J, Altman DG, The PRISMA Group (2009). Preferred Reporting Items for Systematic Reviews and Meta-Analyses: The PRISMA Statement. PLoS Med 6(7): e1000097. 10.1371/journal.pmed1000097. For more information, visit www.prisma-statement.org

The advanced search on PubMed produced a total of 7877 results. The function “Shows similar articles” identified another 158 potentially relevant studies. After reviewing the titles of 8035 studies, 7747 studies were primarily excluded. Of the 288 potentially relevant studies, all studies involving children or animals were excluded secondarily, as well as those studies that used only vital parameters as an indication of anaemia. Therefore, a total of 140 studies were left for full-text review. Finally, 50 further studies were excluded due to: no availability of the full text; letters or comments to the editor; young participants under 18 years of age; the measurement of intraoperative blood loss not primarily addressed and primarily investigation of measurement accuracy of an instrument compared to the laboratory. A total of 90 studies were included in the systematic review (Supplement 1) and six studies were included in the meta-analysis (Fig. 1). The main reason for exclusion was the lack of comparison with a validated reference method.

### Risk of bias assessment

The assessment regarding the risk of bias of the individual studies is presented in detail in Table 1. The risk of bias due to confounders was increased in the majority of the studies. The selection of participants in study groups showed an increased risk of bias in four studies [19–22] and an unclear risk of bias in two studies [23, 24]. The documentation and endpoint assessment were adequately described in most of the studies. A deviation in the intervention phase was not described in any of the studies. The handling of missing data was not adequately described in most studies, so that an unclear risk of bias was found. Double-blinding was found in only a few studies. The risk of selective reporting was low in all except four studies [25–28]. The overall methodological quality of the studies reviewed was rather low and the overall risk of bias across the studies was high.

**Table 1:** Risk of bias of the individual studies

Study	n	Mean fluid (ml)	Sponge	Methode1	Methode2	Correlation	CI_u	CI_o	n	Bias (g Hb)	SE	CI_u
Konig [26]	50	35.3	AMD Ritmed	Triton	Pre-measured	0.94	0.89	0.96	50	− 0.22	0.08	− 0.38
Konig [26]	50	33.5	RF Detect	Triton	Pre-measured	0.94	0.92	0.96	50	− 0.03	0.04	− 0.11
Konig [26]	50	33.5	Cardinal Allegiance	Triton	Pre-measured	0.92	0.89	0.94	50	0.15	0.06	0.04
Konig [26]	57	29.5	NovaPlus	Triton	Pre-measured	0.93	0.91	0.95	57	0.08	0.04	0.00
Holmes [67]	46	668	Mixed	Triton	Rinsing	0.93	0.88	0.96	46	9.00	1.28	6.50
Sharaeh [27]	50	12.7	learCount Medical	Triton	Rinsing	0.92	0.86	0.95	50	6.40	0.89	4.70
Konig [28]	207	250	Canister	Triton (medi	Pre-measured					3.40	0.38	2.60
Konig [68]	50	218	Unclear sponge	Triton	Rinsing	0.91	0.84	0.95	50	3.70	1.41	0.98
Mixed effect meta-analysis						0.9299	0.9155	0.9443		2.65	1.19	0.32

Study	CI_o	Lower limit	SE	CI_u	CI_o	Upper limit	SE	CI_u	CI_o	Comment	p values comparison	p values metaregression (if possible)
Konig [26]	− 0.07	− 1.28	0.08	− 1.43	− 1.13	0.84	0.08	0.68	0.99			
Konig [26]	0.05	− 1.04	0.04	− 1.12	− 0.96	0.98	0.04	0.90	1.06			
Konig [26]	0.26	− 1.15	0.05	− 1.26	− 1.05	1.45	0.06	1.34	1.56			
Konig [26]	0.16	− 0.92	0.04	− 1.00	− 0.84	1.08	0.04	1.01	1.16			
Holmes [67]	11.50	− 7.51	1.28	− 10.00	− 5.00	25.51	1.28	23.00	28.00	Per case		
Sharaeh [27]	8.20	− 5.60	0.87	− 7.30	− 3.90	18.50	0.87	16.80	20.20			
Konig [28]	4.10	− 7.40	0.66	− 8.70	− 6.10	14.20	0.66	12.90	15.50	Correlation lower limit above	0.97	
Konig [68]	6.49	− 15.30	1.40	− 18.00	− 12.50	22.70	1.28	20.00	25.00			
Mixed effect meta-analysis	4.99	− 4.90	1.75	− 8.32	− 1.48	10.60	3.79	3.18	18.02		0.026	0.02

Study	n	Mean fluid (ml)	Sponge	Methode1	Methode2	Correlation	CI_u	CI_o	n	Bias (ml EBL)	SE	CI_u
Holmes [67]	39	180	Mixed	Triton	Rinsing	0.94	0.89	0.97	39	88.00	44.90	− 68.00
Holmes [67]	39	180	Mixed	Gravimetric	Rinsing	0.92	0.86	0.96	39	466.00	53.57	361.00
Sharareh [27]	50	135	ClearCount Medical	Triton	Photometric	0.91	0.85	0.95	50	53.00	7.65	38.00
Sharareh [27]	50	135	ClearCount Medical	Gravimetric	Photometric	0.66	0.47	0.79	50	283.00	24.74	234.00
Doctorvaladan [70]	50	470	RFDetect/Medi-Vac	Triton	Rinsing	0.951	0.915	0.972	50	102.00	15.31	72.00
Doctorvaladan [70]	50	470	RFDetect/Medi-Vac	Gravimetric	Rinsing	0.564	0.339	0.728	50	352.00	58.67	237.00
Doctorvaladan [70]	50	470	RFDetect/Medi-Vac	Visual	Rinsing	0.7	0.523	0.819	50	458.00	31.63	396.00
Konig [28]	207	250	Canister	Triton (medi	Pre-measured					26.80	3.04	20.90
Konig [68]	50	218	Unclear sponge	Triton	Rinsing	0.893			50	13.00	54.86	
Konig [68]	50	218	Unclear sponge	Gravimetric	Rinsing	0.872			50	4.00	170.50	
Konig [68]	44	218	Unclear sponge	Visual	Rinsing	0.483			44	389.00	212.75	
Mixed effect meta-analysis (triton vs. control)						0.93	0.96			57.59	17.20	23.88
Mixed effect meta-analysis (gravimetric vs. control)						0.77	0.93			326.36	63.57	201.65
Mixed effect meta-analysis (visual vs. control)						0.61	0.82			456.51	31.29	395.19

Study	CI_o	Lower limit	SE	CI_u	CI_o	Upper limit	SE	CI_u	CI_o	Comment	p values comparison	p values metaregression (if possible)
Holmes [67]	108.00	− 31.00	10.20	− 51.00	− 11.00	207.00	9.95	187.00	226.00	Per case		
Holmes [67]	571.00	− 171.00	53.57	− 276.00	− 66.00	1103.00	53.57	998.00	1208.00			
Sharareh [27]	68.00	− 53.00	7.91	− 68.00	− 37.00	158.00	7.65	143.00	173.00			
Sharareh [27]	331.00	− 51.00	24.74	− 100.00	− 3.00	616.00	24.74	568.00	665.00			
Doctorvaladan [70]	132.00	− 105.00	15.31	− 135.00	− 75.00	309.00	15.31	279.00	339.00			
Doctorvaladan [70]	467.00	− 441.00	58.67	− 556.00	− 326.00	1145.00	58.67	1030.00	1260.00			
Doctorvaladan [70]	520.00	31.00	31.63	− 31.00	93.00	886.00	31.63	824.00	948.00			
Konig [28]	32.80	− 58.70	5.26	− 69.00	− 48.40	112.40	5.26	102.10	122.70	Correlation lower limit above	0.97	
Konig [68]												
Konig [68]												
Konig [68]												
Mixed effect meta-analysis (triton vs. control)	91.30	− 60.21	14.08	− 87.80	− 32.62	195.79	41.87	113.74	277.85		0.008	0.23
Mixed effect meta-analysis (gravimetric vs. control)	450.86	− 216.68	115.06	− 442.19	8.84	951.33	171.33	615.53	1287.14		< 0.0001	
Mixed effect meta-analysis (visual vs. control)	517.83	31.00	31.63	− 30.99	92.99	886.00	31.63	824.01	947.99		< 0.0001	

Risk of bias of the individual studies are presented. low RoB “++” (study is comparable to a randomized study); moderate RoB “+” (study correctly performed represents a non-randomized study); significant RoB “−” (study has some serious problems); critical RoB “−−" (study has too many serious problems); unclear RoB “?” (due to lack of information no assessment is possible)

### Used methods within surgery or obstetrics

**Fig. 2:**
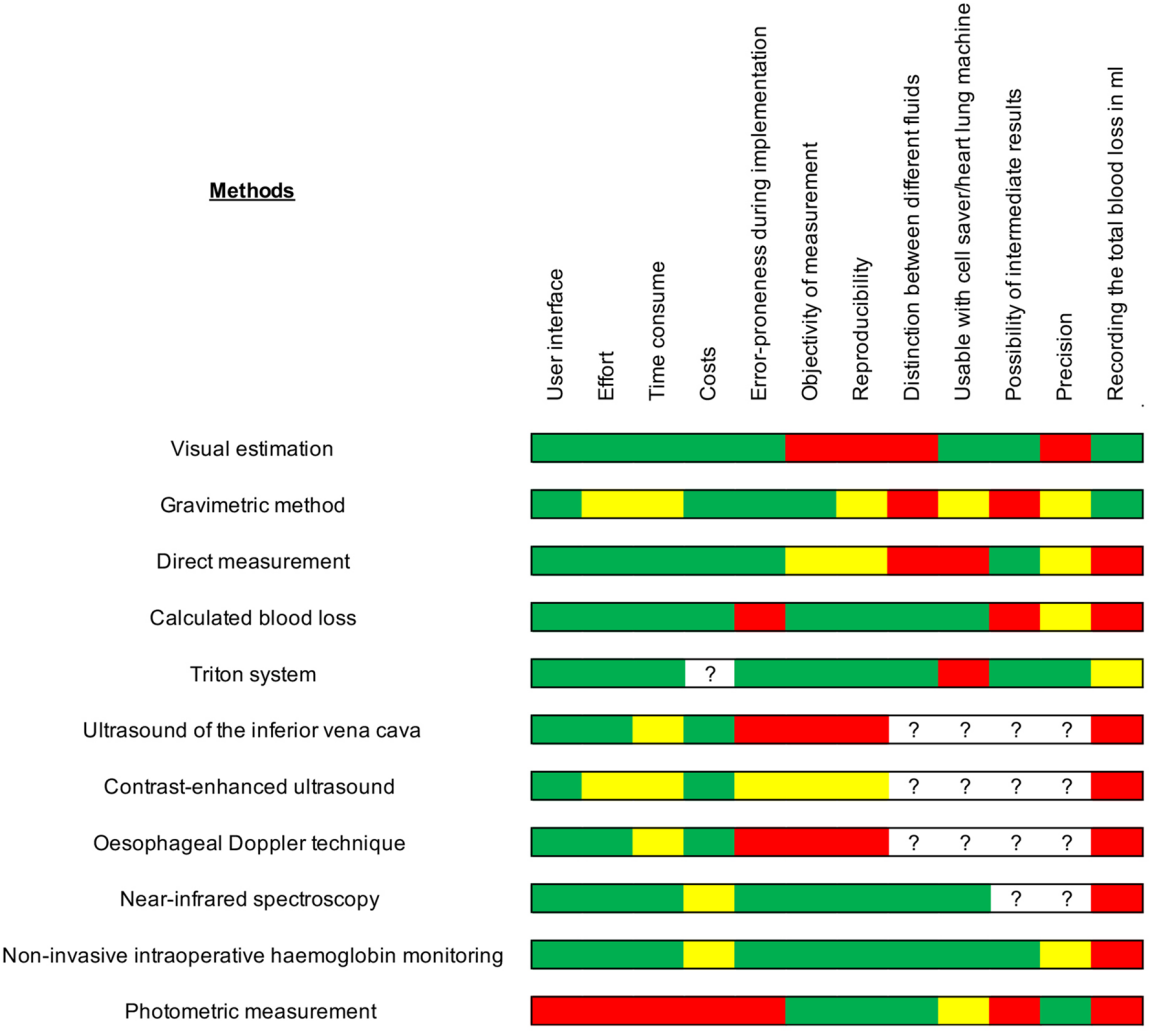
Pros and cons of blood loss estimation methods. Pros and cons of blood loss estimation methods are presented. Red = extremely poor, yellow = moderate; green = excellent

Based on the studies, we examined the methods according to various criteria. The advantages and disadvantages of the individual methods are summarized in Fig. 2.

#### Visual estimation

The visual estimation of blood loss by clinicians is not only one of the most widely used methods, but also the most examined one. This includes the estimation of blood volumes in sponges and suction containers but also the recording of external blood losses. Forty-eight studies dealt with the accuracy and improvement of visual assessment and 29 of these were performed in obstetrics. The study results are heterogeneous, so there are different results about the influence of different factors of professional experience, gender, age on the accuracy of the estimate. Even the use of pictograms and other measures does not lead to clear improvements (for a detailed description of the various studies, please refer to Supplement 2) [29–39].

#### Gravimetric

The gravimetric method is an indirect measurement of blood loss. Blood loss can be deduced by weighing the surgical material contaminated with blood and subtracting the dry weights. By summing up the measured weight of the blood and estimating the amount (ml) of mixed liquids (e.g. blood, rinse liquid) in the suction container, the blood loss can be calculated with a conversion of 1 g = 1 ml blood [38, 66]. The study results for gravimetric methods show a higher degree of correlation but are nevertheless heterogenic. Especially the factor of increased dilution by amniotic fluid or rinsing are relevant factors that lead to inaccuracy in the calculation [27, 67, 68](for a detailed description of the various studies, please refer to Supplement 3).

#### Direct measurement

The direct measurement of blood loss is a simple and long-established method that is mainly used in the field of obstetrics. Nine studies focused on calibrated collection bags specially designed for vaginal deliveries. The collector bag is placed under the woman's buttocks immediately after the birth of the child and collects all mixed liquids (e.g. blood or amniotic fluid). At the bottom of the plastic foil there is a calibrated collector bag with a scale on which the current blood loss can be read. This method is easy to use and, especially in resource-poor areas, in combination with a visual assessment can somewhat improve the quantification of the total blood volume, e.g. during a birth. However, study results still show significant deviations from real blood volume when used [71–75] (for a detailed description of the various studies, please refer to supplement 4).

#### Calculated blood loss

Currently, various mathematical approaches are used in clinical practice to evaluate blood loss. To calculate the most exact intraoperative blood loss, the formula has been modified over time. Thus, three different formulas are used in the literature to calculate the total blood volume of patients. The Moore formula [75, 76], which, like Nadler's formula [75–81] takes into account height, weight and sex for the calculation, and the ICSH formula [75, 76], which uses gender and body surface area to calculate the total blood volume. The formula according to Nadler was used most often in the literature. All blood loss estimation formulas showed a significant tendency to overestimate blood loss. (For a detailed description of the various studies, please refer to supplement 5).

#### Colorimetric blood loss estimation

A smartphone application (Triton™) developed by Gauss Surgical Inc. is able to calculate blood loss by taking photographs of used surgical gauze and canisters. The colorimetric technique analyses photographic and geometric information from relevant areas, with the aim of automatically filtering out the effects of non-blood components mixed in each sponge and canister and calculating the Hb mass present in the gauze or canister from the image. By entering the preoperative Hb-level, the blood loss can then be calculated. In the studies analyzed, high degrees of correlation with the reference blood volumes were found [22, 24, 26–28, 39, 67, 68, 70, 83] (For a detailed description of the various studies, please refer to supplement 6).

#### Miscellaneous methods

Other methods for the intraoperative recording of blood loss are rarely described and have not yet been sufficiently tested. Ultrasound of the inferior vena cava [20, 84, 85], contrast enhanced ultrasound (CEUS) [86], hemodynamic esophageal Doppler monitoring [19], near-infrared spectroscopy [87] or continuous non-invasive intraoperative Hb monitoring [23, 88, 89] for intraoperative detection of blood loss have been investigated. None of these methods represents a valid technique for blood loss detection. (For a detailed description of the various studies, please refer to supplement 7).

### Meta-analysis

Six studies [26–28, 67, 68, 70] used Hb assay as a validated reference method and Bland Altman results were available for meta-analysis. The mixed effect meta-analysis showed the highest correlation to the reference for colorimetric methods (0.93 95% CI 0.91–0.96), followed by gravimetric (0.77 95% CI 0.61–0.93) and finally visual methods (0.61 95% CI 0.40–0.82) (Table 2). The bias for estimated blood loss (ml) was lowest for colorimetric methods (57.59 95% CI 23.88–91.3) compared to the reference, followed by gravimetric (326.36 95% CI 201.65–450.86) and visual methods (456.51 95% CI 395.19–517.83) (Table 2).

**Table 2:** Results of meta-analysis

Risk of bias of the individual studies are presented. low RoB “++” (study is comparable to a randomized study); moderate RoB “ + ” (study correctly performed represents a non-randomized study); significant RoB “−” (study has some serious problems); critical RoB “−−” (study has too many serious problems); unclear RoB “?” (due to lack of information no assessment is possible)

**Fig. 3:**
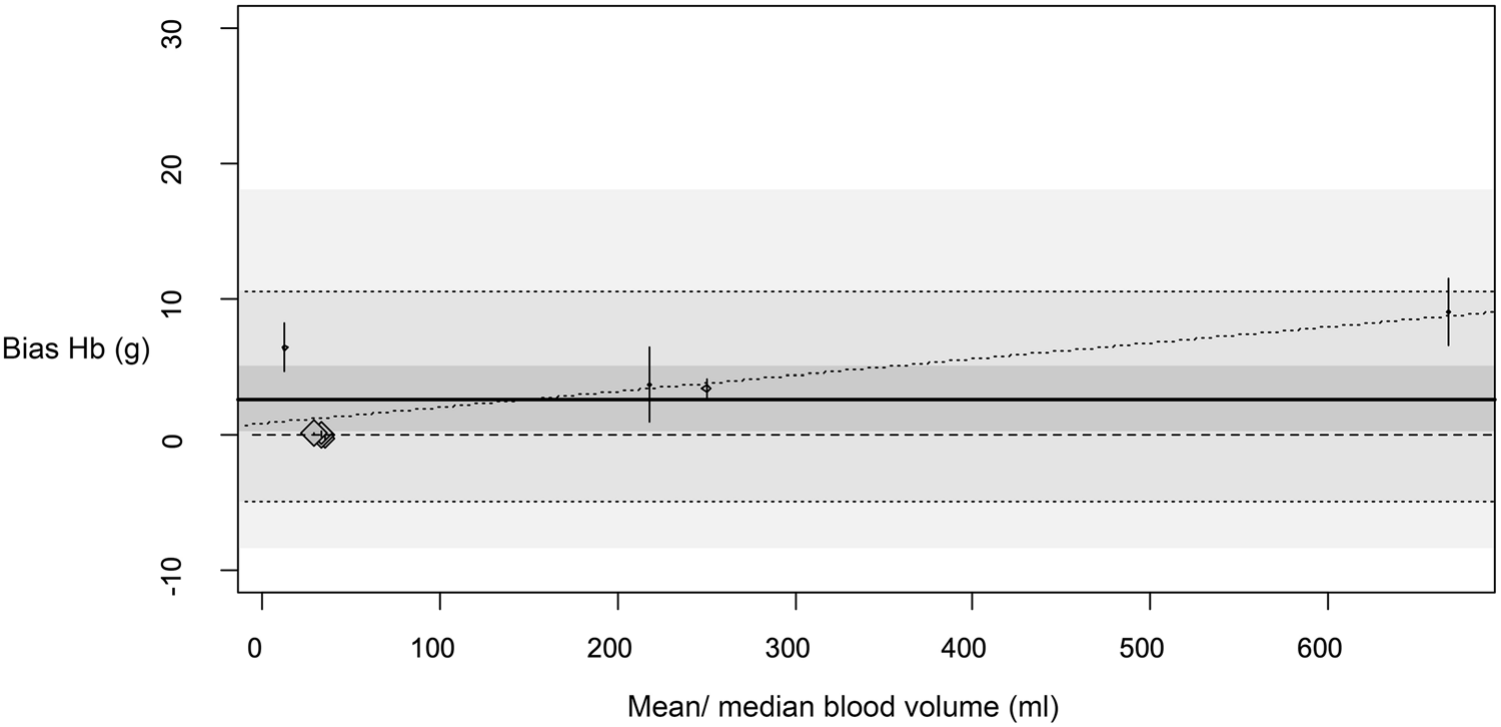
Bias of Hb [g] measurements with colorimetric method. Bias of Hb [g] measurements with colorimetric method compared to reference. Diamonds show the bias estimates from the single studies (size of diamonds is inversely related to standard error). Small vertical bars show confidence intervals of bias in the single studies. The darkest grey shaded area is the overall confidence interval for the estimated bias. The thick joined line shows the overall bias estimate. The medium shaded area reaches from the overall estimate of the lower tolerance limit to the overall estimate to the upper tolerance limit, both limits are also indicated by a dotted line. The lightest shaded area reaches from the lower confidence interval limit of the lower tolerance limit to the upper confidence interval limit of the upper tolerance limit

Figure 3 shows the bias of the blood loss [ml] measurements with the colorimetric method compared to the reference. The overall bias estimate is significantly different from zero (p = 0.0008). There is no significant increasing trend toward a higher bias for higher mean or median blood loss used in the individual studies (p = 0.2317). The results of the meta-regression analysis show significant heterogeneity I^2^ = 87.9%, p < 0.0001 and R^2^ = 15.24% of the heterogeneity considered in the meta-regression model.

**Fig. 4:**
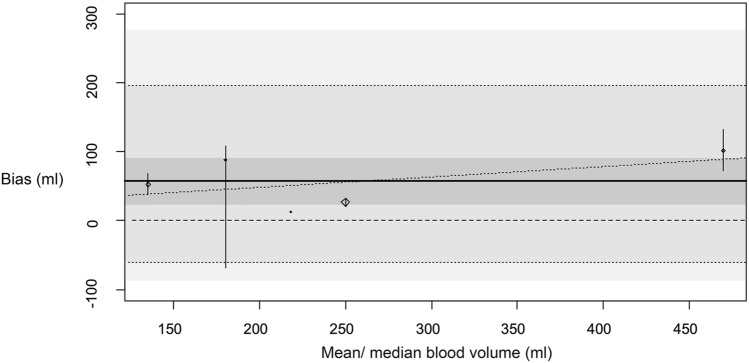
Bias of blood volume [ml] estimates with colorimetric method. Bias of blood volume [ml] estimated by colorimetric method compared to reference. Diamonds show the bias estimates from the single studies (size of diamonds is inversely related to standard error). Small vertical bars show confidence intervals of bias in the single studies. The darkest grey shaded area is the overall confidence interval for the estimated bias. The thick joined line shows the overall bias estimate. The medium shaded area reaches from the overall estimate of the lower tolerance limit to the overall estimate to the upper tolerance limit, both limits are also indicated by a dotted line. The lightest shaded area reaches from the lower confidence interval limit of the lower tolerance limit to the upper confidence interval limit of the upper tolerance limit

The overall bias of colorimetric estimated blood loss is significantly different from 0 (p = 0.026). The bias of the Hb mass [g] measurements with the colorimetric system compared to the reference is shown in Fig. 4. The correlation for colorimetric Hb measurement correlated strong (0.930 95% CI 0.96–0.94) with reference Hb. There is a significantly increasing trend toward higher distortion for higher mean or median blood volume used in the individual studies (p = 0.020). The results of the meta-regression analysis still show a significant heterogeneity I^2^ = 99.9%, p < 0.0001 and R^2^ = 53.26% of the heterogeneity considered in the meta-regression model.

## Discussion

We were able to show in our meta-analysis that the colorimetric blood estimation provides a strong correlation to the reference blood volume. In contrast, gravimetric method or visual estimation shows only a medium correlation. It was shown that the bias for blood loss in the colorimetric system is significantly lower than in the other methods.

Visual estimation is the most widely used method for recording intraoperative blood loss and can be performed directly in the operating theatre without the need for additional equipment. However, regardless of specialty, professional experience or education level, visual estimation is not accurate. The estimation of lost blood volume depends on the physicians and is hard to reproduce. Especially in scenarios with larger bleeding the visual estimation often fails. A clear tendency to over- or underestimation cannot be detected, but either one can lead to wrong decisions in patient care.

The gravimetric method considers external blood loss. Frequently used conversion in literature “1 g equals 1 ml of blood” is only an approximation. Blood, depending on the current Hct, does not have the same density as water. Both lead to measurement inaccuracies. The calculation of the lost blood volume is a cost-effective way to quickly provide information about the suspected blood loss. Many of the formulas are based on Hb-value or Hct changes and require normovolaemia to calculate the patient's total blood volume. This assumption can lead to measurement inaccuracies. It is known, that the measurement of the Hb-level through blood gas analysis only shows indications of blood loss after adequate volume therapy and is therefore delayed. This can also lead to measurement inaccuracies. The result is independent of the user and easy to apply, especially if a user-friendly program is used for support.

The latest innovative method to detect blood loss is the colorimetric estimation technique. Our meta-analysis revealed a strong correlation to reference blood volume. The overall bias estimate shows that the systematic bias is within the 30 g Hb mass, which was defined as the clinically relevant limit. Thirty grams Hb-mass corresponds to approximately 1/10 of a whole blood unit. External blood loss cannot be taken into account by the system. If a cell saver or the heart–lung machine is involved, the system can only be used partially because the calibration of the canisters required for the colorimetric system is not sterile.

### Limitations

We have identified several limitations for this review and meta-analysis. The selected reference methods differ widely and are usually not considered to be sufficiently valid themselves. There is no gold standard reference for recording intraoperative blood loss. The sample size of the individual studies was mostly small. Most of the studies were prospective observational studies without double-blinding and control group. The risk of bias and heterogeneity for the individual studies was high. Although many studies are available in this area, one weakness of most studies is that no validated reference was used for comparison. Many studies compare themselves with other equally inaccurate methods. This underlines the need for more high-quality large-scale studies in this area.

## Conclusion

The recording of intraoperative blood loss plays a very central role in the daily routine of clinicians. Based on these estimations, patient’s treatment and transfusion decisions are made. Consequently, for the patient safety, we should aim fort the highest possible accuracy of measurement. Visually and gravimetric blood loss estimation measurements show a high degree of bias, so its usage cannot be recommended. Colorimetric technology offers real-time measurement and has a high degree of correlation.

## Electronic supplementary material

Below is the link to the electronic supplementary material.

Supplementary file1 (PDF 103 kb)

Supplementary file2 (DOCX 103 kb)

Supplementary file3 (DOCX 38 kb)

Supplementary file4 (DOCX 30 kb)

Supplementary file5 (DOCX 51 kb)

Supplementary file6 (DOCX 40 kb)

Supplementary file7 (DOCX 38 kb)

## Abbreviations

Acrobat-NRSI: A Cochrane Risk of Bias Assessment Tool: for Non- Randomized Studies of Interventions
CEUS: Contrast-enhanced ultrasound
cSO_2_: Hb saturation of left frontal lobe
FET: Feature Extraction Technology
Hb: Haemoglobin
HbD: Oxygenation index
Hct: Hematocrit values
ICSH: International Council for Standardization in Haematology
OSTHEO: Orthopedic Surgery Transfusion Haemoglobin European Overview
PBM: Patient Blood Management
PPH: Postpartum hemorrhage
pSO_2_: Hb saturation of left calf
QBL: Quantification of Blood Loss
RBC: Red blood cell units
RoB: Risk of Bias
SD: Standard deviation
USD: US Dollar
