# Supplementary material for: Comparison of common perioperative blood loss estimation techniques: a systematic review and meta-analysis
Source: J Clin Monit Comput. 2020 Aug 19;35(2):245–58. doi: 10.1007/s10877-020-00579-8 (PMC7943515; doi:10.1007/s10877-020-00579-8)
Supplement: Supplementary file 1 — Supplementary file1 (PDF 103 kb) [file 10877_2020_579_MOESM1_ESM.pdf]

Author(s) (YearPublished)	Study Design	Study topic	Included Groups (n)	Outcomes	Link
**Published systematic Reviews**					
Schorn et al (2010)	SR	Blood loss measurement in obstetrics	46 publications	-identified methods: visual estimation (EBL), direct measurement (DM), gravimetric (gEBL), photometry, calculated blood loss (CBL), measurement of the change in the diameter of the inferior vena cava (IVCd), tagging red blood cells (RBCs), serum-specific gravity, central venous blood saturation	https://pubmed.ncbi.nlm.nih.gov/20129226/
Gabel et al (2012)	SR	Blood loss measurement in obstetrics	No information	-identified methods: EBL, acid hematin method (AHM), gEBL, DM, laboratory tests	https://pubmed.ncbi.nlm.nih.gov/22548283/
Hancock et al (2015)	SR	Blood loss measurement in obstetrics	36 publications	-accuracy of EBL; methods to improve EBL	https://pubmed.ncbi.nlm.nih.gov/26415952/
**Visual Estimation: Testing in Simulations**					
Meiser et al (2001)	P-SB-OS	Accuracy of EBL; influencing factors (IF)	SG(36)	-over- and underestimates by factor 2 and 3<br>-there were more underestimates<br>-diluted blood was often overestimated<br>-gender, age and professional experience of the participants did not show any influence<br>-anaesthetists estimated on average significantly more than the surgeons	https://pubmed.ncbi.nlm.nih.gov/11220251/
Beer et al (2005)	P-SB-OS	Accuracy of EBL; IF	SG (32)	-primarily underestimation, the larger the volume, the more it was underestimated<br>- no significant difference in the medical professions	https://pubmed.ncbi.nlm.nih.gov/15807956/
Bose et al (2006)	P-SB-OS	Identification of the areas of greatest discrepancy between EBL and actual blood loss (ABL)	SG (103)	-significant underestimation of large external blood losses<br>-all professional groups underestimated the capacity of a large swab<br>-anaesthetists were the most accurate estimators	https://pubmed.ncbi.nlm.nih.gov/16907938/
Buckland et al (2007)	P-SB-OS	Accuracy of EBL	SG (88)	-blood in containers were more accurately estimated than blood on clothes or bandages<br>-lower blood volumes were estimated more accurately than higher volumes	https://pubmed.ncbi.nlm.nih.gov/17320496/
McConnell et al (2007)	P-SB-OS	Influence of colour, volume of the fluid and experience on the accuracy of EBL	SG (152)	-clinicians with less experience tended to estimate better than clinicians with more experience<br>-tendency to overestimate is significantly greater for the larger volumes compared to the smaller volumes<br>-colour had no significant effect on the estimation by the physicians, but for the nursing group	https://pubmed.ncbi.nlm.nih.gov/17451955/
Yoong et al (2010)	P-SB-OS	Accuracy of EBL; consistency of estimations	SG (47)	-general tendency to overestimate<br>-no significant difference between the professional groups<br>-highest inaccuracy for small volumes<br>-reliability of the test retests was poor for the larger volumes, but statistically acceptable for the smaller volumes	https://pubmed.ncbi.nlm.nih.gov/19434419/
Adkins et al (2014)	P-SB-OS	Accuracy of EBL; IF	SG (91)	-no difference in the accuracy of the EBL based on years of anaesthesia experience, training, gender, ethnicity, or education	https://pubmed.ncbi.nlm.nih.gov/25167610/
Parayre et al (2015)	Multicentre CSS	Accuracy of EBL made by student midwives; intra-observer agreement	SG (463)	-35.34% of the answers were correct<br>-reproducibility rate for EBL was moderate<br>-percentage of accurate answers was higher for low volumes and higher for high volumes	https://pubmed.ncbi.nlm.nih.gov/26233835/
Rothermel et al (2016)	P-SB-OS	Accuracy of EBL; IF	SG (60)	-total estimates were significantly inaccurate<br>-27% of the participants showed agreement in over- or underestimating blood loss<br>-no correlation between subject area, years of experience or confidence in the ability to estimate blood loss consistently or accurately	https://pubmed.ncbi.nlm.nih.gov/27544540/
Pranal et al (2018)	Multicentre CSS	Accuracy of EBL made by (student) midwives; intra-observer agreement	SG (1041)	-34.1% of the estimations were correct, 37.2% underestimated the volume and 28.7% overestimated<br>-the intra-observer reproducibility was better at higher volumes<br>-the students underestimated the blood loss more often than the midwives	https://pubmed.ncbi.nlm.nih.gov/29348050/
Kollberg et al (2019)	P-SB-OS	Accuracy of EBL	SG (163)	-provided both over- and underestimates<br>-underestimates dominated and tended to increase with larger blood volumes<br>- participants estimated an average error of 30% regardless of profession and years of experience	https://pubmed.ncbi.nlm.nih.gov/31587711/
**Visual Estimation: operation Theatre**					
Howe et al (2003)	RCA	EBL in a formula for predicting postoperative haemoglobin (Hb) concentration vs. actual postoperative Hb concentration	SG (198)	-EBL vs. change in Hb value gave a correlation coefficient of 0.189 and a p value of 0.008	https://pubmed.ncbi.nlm.nih.gov/12812382/

McCullough et al (2004)	RCA	CBL based on pre- and postoperative Hb levels vs. EBL	SG (52)	-no significant difference between the estimates of anaesthetists and surgeons<br>-significant difference between CBL and EBL	https://pubmed.ncbi.nlm.nih.gov/14730159/
Kavle et al (2006)	P-CS	AHM vs. EBL of midwifes	SG (158)	-average difference between the midwives' EBL and the laboratory measurements was 4.90 ml<br>-inaccuracy of midwives was higher with blood loss of 200 ml and more	https://pubmed.ncbi.nlm.nih.gov/16919628/
Larsson et al (2006)	P-CS	AHM vs. EBL	SG (55)	-EBL showed a tendency to overestimation compared to AHM<br>-in vaginally delivered women there was no correlation between EBL and AHM (r=0.13)<br>-in women delivered by elective caesarean section the correlation was moderate (r=0.55)	https://pubmed.ncbi.nlm.nih.gov/17260220/
Stafford et al (2008)	P-CS	EBL vs. CBL (reference method)	SG (677)	-CBL was significantly different from EBL<br>-the higher the CBL the greater the deviation of the EBL	https://pubmed.ncbi.nlm.nih.gov/18639209/
Wangwe et al (2012)	P-CS	Accuracy in the diagnosis of postpartum haemorrhage (PPH) by EBL vs. change in haematocrit (HCT)	SG (426)	-change in HCT in the diagnosis of PPH proved to be more accurate, specifically with high positive predictive values (PPV) compared to EBL	https://pubmed.ncbi.nlm.nih.gov/26591737/
Guinn et al (2013)	P-R-CT	EBL vs. non-invasive Hb measurement	SG (60)	-mean EBL exceeded the measured blood loss by an average of 40%	https://pubmed.ncbi.nlm.nih.gov/23438094/
Ram et al (2014)	P-R-CT	EBL vs. modified gross formula using HCT values	SG (140)	-with a low average blood loss, the EBl accuracy is high<br>-when the average blood loss exceeded 500 ml, the accuracy rate decreased significantly	https://pubmed.ncbi.nlm.nih.gov/25098850/
Conner et al (2015)	P-CS	EBL vs. CBL and HCT drop	SG (4804)	-weak correlation between EBL and CBL was found in all measurements	https://pubmed.ncbi.nlm.nih.gov/26007310/
Lertbunnaphong et al (2016)	P-CS	EBL vs. DM	SG (286)	-in terms of accuracy, the EBL proved to be inaccurate<br>-tendently was rather underestimated, with low agreement (27.6%)<br>-two thirds of cases of immediate PPH (65.4%) were misdiagnosed by EBL	https://pubmed.ncbi.nlm.nih.gov/27353510/
Withanathantrige et al (2016)	Second analyse of a R-C-CT	combination of DM and gEBl (MBL) vs. EBL vs. CBL using the HB drop	SG (156)	-EBL by the anaesthetists had the best intraclass correlation (0.713) and limits of agreement with MBL	https://pubmed.ncbi.nlm.nih.gov/27451396/
Budair et al (2017)	P-CS	EBL vs. combination of gEBL and subtraction of the total amount of the used rinsing solution from the total volume in the suction bag	SG (55)	-EBL by anaesthetists significantly more accurately than surgeons<br>- no significant correlation between the ABL and EBL by surgeons (consistently underestimated)	https://pubmed.ncbi.nlm.nih.gov/27829730/
Gluck et al (2017)	R-CS	EBL vs. CBL; identify independent risk factors for severe underestimation and overestimation	SG (3655)	-in 11.5% of cases, a severe underestimation of blood loss was found, the individuals in this group had a significantly higher body mass index (BMI), lower rates of previous caesarean sections, higher rates of urgent caesarean sections and general anaesthesia<br>-in 33.2% of the cases a serious overestimation of blood loss was found, the persons in this group had a significantly lower gestational age, higher rates of previous caesarean sections, multiple pregnancies, intra-abdominal adhesions and lower rates of urgent caesarean sections and general anaesthesia	https://pubmed.ncbi.nlm.nih.gov/28879437/
Hamm et al (2018)	P-OS	Would the Quantification of blood loss calculator (QBL) improve the prediction of HB-waste 12 hours after birth compared to EBL?	SG (583)	-for all measurements, QBL did not predict the HB drop more accurately than the EBL	https://pubmed.ncbi.nlm.nih.gov/28838005/
Serapio et al (2018)	P-C-COS	EBL vs. MBL	SG (371)	-measurement by MBL was about twice as high as EBL<br>-participants consistently and significantly underestimate the blood loss	https://pubmed.ncbi.nlm.nih.gov/29410259/
Ulusoy et al (2018)	P-CT	EBL vs. gEBL	SG (65)	-if the blood loss during the operation was high, the difference between the surgeon's and anaesthetist's estimates was also higher	https://pubmed.ncbi.nlm.nih.gov/29705298/
Anya et al (2019)	P-DB-C-COS	EBL vs. continuous non-invasive HB monitoring with a HemoCue	SG (60)	-the bias between the methods was not significant (45.25 ml)<br>-the discrepancy between the two methods increased when the blood loss was more than 500 ml	https://pubmed.ncbi.nlm.nih.gov/30860196/
**Visual Estimation: Tools and Training**					
Dildy et al (2004)	P-SB-OS	Are didactic sessions an improvement of EBL?	SG (53)	-there was a significant reduction in errors<br>-professional experience had no influence on the EBL<br>-blood loss tended to be overestimated at low volumes and underestimated at high volumes	https://pubmed.ncbi.nlm.nih.gov/15339775/
Sukprasert et al (2006)	R-C-CT	Are didactic sessions an improvement of EBL?	IG (45)<br>CG (45)	-gave significant improvement after the training<br>-before the training there was a tendency to overestimate and afterwards there was no tendency	https://pubmed.ncbi.nlm.nih.gov/17726810/
Maslovitz et al (2008)	P-SB-OS	Improvement of EBL through simulation-based practical training with dummies?	regular (126)<br>intervall (24)	-accuracy of EBL improves when the intermediate blood loss is estimated at regular intervals and then added together<br>-residents underestimated the blood loss by an average of 49%<br>-midwives scored better with an underestimation of 40% on average	https://pubmed.ncbi.nlm.nih.gov/18720041/

Merlin et al (2009)	O-CRS	visual aid for external blood loss: MAR method	SG (74)	-one fist surface is equivalent to 20 ml of blood on the floor<br>-for smaller volumes, the mean error has decreased by 76% from the average compared to EBL<br>-larger volumes, the mean error has decreased by 40% from the average	https://pubmed.ncbi.nlm.nih.gov/19931755/
Toledo et al (2010)	P-SB-OS	Are live or web-based didactic sessions an improvement of EBL?	live-based (231)<br>web-based (141)	-the average improvement between the results before and after the training was 34%<br>-this improvement did not differ significantly between the live-based and the web-based sessions	https://pubmed.ncbi.nlm.nih.gov/20035920/
Toledo et al (2012)	P-SB-OS	How much knowledge remains after 9 months of web-based training?	web-based (44)	-the median estimation error decreased to -34.6% compared to the pre-training error of +47.8%<br>-the 9-month error was significantly less accurate than the error of -13.5% that occurred immediately after training	https://pubmed.ncbi.nlm.nih.gov/22228284/
Cheerranichanunth et al (2012)	P-DS	Using blood loss pictograms for EBL	SG (49)	-use of the pictogram leads to a significant increase of accuracy (from 30.9% to 61.8%)<br>-the EBL error decreased from 69.1% to 38.2%	https://pubmed.ncbi.nlm.nih.gov/22612010/
Al-Kadri et al (2014)	OS	Are didactic sessions an improvement of EBL?	SG (123)	-general tendency to underestimate<br>-significant improvement of EBL after training	https://pubmed.ncbi.nlm.nih.gov/24646156/
Zuckerwise et al (2014)	P-SB-OS	Effectiveness of pocket cards with comparative images to improve EBL	SG (151)	-a significant improvement of EBL for all types of providers after intervention in 4 out of 6 stations<br>-experience had no influence on the accuracy of EBL	https://pubmed.ncbi.nlm.nih.gov/24785850/
Golmakani et al (2015)	DS	GEBL vs. national guideline (use of visual aids and pictograms)	SG (112)	-significant difference between the gEBL and that based on the national guideline	https://pubmed.ncbi.nlm.nih.gov/26257803/
Ali Algadiem et al (2016)	CSS	Development of a visual guideline for the EBL on sponges	none	-the absorption capacity of different swabs was determined<br>-the degree of absorption reduction was also determined for swabs with saline solution	https://pubmed.ncbi.nlm.nih.gov/27626017/
Kordi et al (2016)	P-R-CT	Web-based vs. simulation-based vs. conventional training	web-based (35)<br>conventional (35)<br>simualtion-based (35)	-accuracy of EBL after training increased significantly in all groups<br>-the mean score of blood loss estimation after training did not significantly different between the groups	https://pubmed.ncbi.nlm.nih.gov/27500175/
Brooks et al (2017)	P-SB-OS	Use of a visual aid in addition to a collector Bag	SG (69)	-in all cases, participants' estimates were significantly more accurate when the collection bag with the baby scale was used without visual aids	https://pubmed.ncbi.nlm.nih.gov/28429722/
Khadilkar et al (2016)	P-R-CT	Usefulness of training modules and clot conversion factor	DM (100)<br>gEBL(50)<br>EBL (54)	-age and professional experience had no influence on the extent of the estimation error<br>-obstetricians and nurses underestimated the blood loss, whereas anaesthetists more often either overestimated or almost accurately estimated the blood loss<br>-simple educational programs can improve the underestimation of blood loss	https://pubmed.ncbi.nlm.nih.gov/27651622/
Homcha et al (2017)	P-SB-OS	Creation of pictorial guidelines	SG (46)	-use of the guidelines has made the EBL more accurate<br>-after the intervention, the number of participants who estimated within 5% of the actual volume increased from 7% before the intervention to 24%	https://pubmed.ncbi.nlm.nih.gov/28697055/
Mbachu et al (2017)	P-OS	Are didactic sessions an improvement of EBL?	SG (144)	-significant differences in the mean error of EBL before and after the training session	https://pubmed.ncbi.nlm.nih.gov/28236647/
Willcox et al (2017)	P-OS	Tool to improve the EBL: SAPHE mat	SG (36)	-EBL were within 100 mL in 69% of cases and within 200 mL in 97% of cases	https://pubmed.ncbi.nlm.nih.gov/27456310/
Yeung et al (2017)	P- paired-control-S	Development of a nomogram for external blood loss	SG (61)	-the percentage error of EBL was 43%, while the percentage error was 23% when using the nomogram	https://pubmed.ncbi.nlm.nih.gov/29108791/
Nelson et al (2018)	P-SB-OS	Pictographic tool to improve EBL	SG (81)	-use of the pictogram led to significant overestimates<br>-the postoperative change in HB correlated better with the EBL of participants without pictogram	https://pubmed.ncbi.nlm.nih.gov/28922304/
**Gravimetric Method**					
Ambardekar et al (2014)	P-R-CT	GEBL vs. DM	DM (45)<br>gEBL (450)	-the mean blood loss was higher with DM than the gEBL	https://pubmed.ncbi.nlm.nih.gov/25128176/
Lilley et al (2015)	P-SB-OS	GEBl vs. EBL	SG (356)	-the mean percentage error of the gEBl was 4.0 ± 2.7% compared to the EBL with a mean percentage error of 34.7 ± 32.1%<br>-the correlation coefficient between the measured blood loss and the corrected HB drop was 0.77	https://pubmed.ncbi.nlm.nih.gov/25433576/
Atukunda et al (2016)	RCA of a R-CT	GEBL vs. HB drop for diagnosis of PPH	SG (1140)	-gEBL generally had a low sensitivity for the<br>-gEBL has a high PPV (>85%) in high prevalence settings when the blood loss exceeds 750 ml	https://pubmed.ncbi.nlm.nih.gov/27050823/
Ladouceur et al (2019)	Quality improvement project	Survey after implementation of the gEBl	SG (60)	-69% (n = 18) felt that the gEBL was the best approach<br>-8% (n = 2) stated that it was not useful	https://pubmed.ncbi.nlm.nih.gov/31682790/
**Direct Measurement**					
Strand et al (2003)	P-CS	Use of cholera beds during vaginal births	SG (814)	-significant reduction of PPH was found when using the cholera beds compared to the controls	https://pubmed.ncbi.nlm.nih.gov/14620424/

Patel et al (2006)	R-C-CT	Photospectrometry (reference method) vs. DM vs. EBL	EBL(61)<br>DM (62)	-EBL 33% lower than DM<br>-Correlation between the DM and the photospectrometric measurement was 0.92	https://pubmed.ncbi.nlm.nih.gov/16626718/
Toledo et al (2007)	R-CRS	Improvement of EBL through the calibration of the collector bags?	first noncallibrated (53)<br>first callibrated (53)	-no difference in the accuracy of EBL based on professional group, level of education or years of experience<br>-the addition of calibration marks significantly improves the accuracy of estimation in conical vaginal drapes	https://pubmed.ncbi.nlm.nih.gov/18042876/
Zhang et al (2010)	Cluster-R-CT	Number of PPH diagnoses identified with EBL vs. direct measurement	IG (11037)<br>CG (14244)	-severe PPH´s was not reduced by the use of a collection bag compared to EBL of PPH	https://pubmed.ncbi.nlm.nih.gov/20123835/
Tixier et al (2011)	P-CS	Validation of DM for the diagnosis of PPH (confirmation of PPH diagnosis by HB and HCT drop)	SG (122)	-threshold of 500 ml, the sensitivity of the pouch was 6.7% and the specifity was 94.2%; the PPV was 66.7% and the negative predictive value (NPV) was 94.2%<br>-threshold of 300 ml, sensitivity was 88.9%, specifity was 82.7%; the PPV was 47.0% and the NPV was 97.7%	https://pubmed.ncbi.nlm.nih.gov/19876638/
Legendre et al (2016)	P-SB-OS	Validation of DM in a simulation training	SG (98)	-93% to 98% of the participants were correct in their estimation<br>-low volumes were rather overestimated and higher volumes rather underestimated	https://pubmed.ncbi.nlm.nih.gov/26755071/
Abbaspoor et al (2017)	Analytic-diagnostic-S	Validation of DM for the diagnosis of PPH (confirmation of PPH diagnosis by HB and HCT drop)	SG (100)	- at a threshold value of 500 ml the sensitivity was 80%, specificity was 95. 7%, PPV was 88.9% and NPV was 91.8%	https://pubmed.ncbi.nlm.nih.gov/29595030/
**Calculated Blood loss**					
Hurle et al (2004)	RCA	Formula based on pre- and postoperative HCT values	SG (126)	- 6 of 13 patients with a preoperative HCT under 40% and 14 of 113 with a preoperative HCT over 40% required an allogeneic transfusion of packed erythrocytes	https://pubmed.ncbi.nlm.nih.gov/14963354/
Božičković et al (2011)	P-O-CT	Usability of the HB concentration drop in CBL (reference method: DM)	SG (14)	-a linear correlation with a strong correlation coefficient (0.90809) was found only when the transfused blood volume was taken into account	https://pubmed.ncbi.nlm.nih.gov/21327754/
Milosevic et al (2011)	P-CT	CBL based on the postoperative decrease of HB and HCT levels vs. DM	SG (1487)	-confirms the applicability of a one-compartment model, based on the decrease in HB values, for CBL	https://pubmed.ncbi.nlm.nih.gov/21553340/
Stahl et al (2012)	P-R-CT	Blood loss score vs. combination of gravimetric method and spectroscopy	Derivation (50)<br>Validation (50)	-the score, which includes suction fluid volume and HB concentration, explains more the variance of the measured blood loss than the experts' assessment (77% vs. 54%, $p = 0.05$) or the change in HB concentration (77% vs. 11%, $p < 0.0001$)	https://pubmed.ncbi.nlm.nih.gov/22352443/
Gao et al (2015)	RCA	Formula comparison: gross formula, Hb-balance, OSTHEO formula, Hb dilution	SG (245)	-provided large differences in the CBL´s<br>-HB balance method is perhaps the most reliable method for estimating blood loss	https://pubmed.ncbi.nlm.nih.gov/26521781/
Lopez-Picardo et al (2017)	RCA of aDB-R-CT	CBL (using the formulas of Bourke, Gross, Mercuriali, and Camarasa and Lopez-Picardo) vs. gEBL	single dose (35)<br>two dose (35)<br>placebo (35)	-comparing results of the Lopez-Picardo formula and other formulas, a moderate-to-low agreement (in terms of Intra-class correlation (ICCs)) was found except for that of Camarasa (ICC: 0.992)	https://pubmed.ncbi.nlm.nih.gov/28368940/
Kahr et al (2018)	Second analyse of a P-OS	modified Brecher`s formula vs. DM	SG (921)	-with caesarean sections, large blood losses tended to be underestimated and small blood losses tended to be overestimated<br>-high correlation of methods for vaginal births ($p < 0.001$, $r = 0.683$)	https://pubmed.ncbi.nlm.nih.gov/30225686/
Jaramilo et al (2019)	P-OS	the formulae according to Wards, Bourke and Smith, Gross, HB difference, OSTEHO and Lopez-Picardo vs. DM	SG (80)	-examined formulas showed poor agreement with DM<br>-significant distortions led to overestimation for most formulae<br>-best agreement on the formula of López-Picado after 48 hours	https://pubmed.ncbi.nlm.nih.gov/30488961/
**Triton System**					
Holmes et al (2014)	Multicentre P-CT	Triton system vs. gEBL vs. HB extraction assay (reference)	SG (46)	-significant positive linear correlation between Triton System and the reference method (0.93, $p < 0.000$)<br>-Bland-Altman analysis showed a bias of 9.0 g and narrow limits of agreement (-7.5 g to 25.5 g)<br>-Bland-Altman analysis of the gravimetric method showed a bias of 466 ml (overestimation)	https://pubmed.ncbi.nlm.nih.gov/24797122/
Konig et al (2014)	VVS	In vitro evaluation Triton System for sponges; tests of different sponges and light conditions	none	-average total percentage error in measuring HB loss for the Triton system was 12.3% [95% CI 8.2 to 16.4%]<br>-strong positive linear correlation between the values measured by Triton and the actual HB masses over the entire range of intraoperative lighting conditions<br>-Bland-Altman analysis showed a bias of 0.01 g [95% CI -0.03 to 0.06 g] HB mass per sponge	https://pubmed.ncbi.nlm.nih.gov/24806138/
Sharareh et al (2015)	P-SB-OS	Triton system vs. gEBL vs. HB extraction assay (reference)	SG (50)	-significant positive correlation between the Triton system and photometric analysis for HB mass and blood loss at 0.92 and 0.91	https://pubmed.ncbi.nlm.nih.gov/26401167/
Doctorvaladan et al (2017)	P-SB-OS	Triton system vs. gEBL vs. EBL vs. HB extraction assay (reference)	SG (50)	-the correlation between the Triton System and the reference method was more predictive ($r=0.951$) than either EBL ($r=0.700$) or gEBL($r=0.564$)	https://pubmed.ncbi.nlm.nih.gov/28497007/

Thurer et al (2017)	RCA	accuracy of the Triton system evaluated by predicting the postoperative HB value vs. EBL	SG (167)<br>CG (100)	-formula with the Triton measured blood loss was a better predictor of the actual postoperative HB value of day 1 than the same formula with the EBL	https://www.longdom.org/open-access/accurate-measurement-of-intraoperative-blood-loss-improves-prediction-ofpostoperative-hemoglobin-levels-2155-6148-1000743.pdf
Konig et al (2018)	VVS	In vitro evaluation Triton System for canisters; tests of different light conditions	none	-HB mass bias correlated significantly with haemolysis level and total canister volume<br>-high agreement between the Triton System and the reference method over all lighting conditions	https://pubmed.ncbi.nlm.nih.gov/29239963/
Konig et al (2018)	P-SB-OS	Triton system vs. gEBL vs. EBL vs. HB extraction assay (reference)	SG (50)	-the correlation between reference and Triton remained high and the systematic distortion low throughout the measurement period<br>-correlation between EBL and the reference measurements exists but a greater dispersion of data points<br>-correlation between gEBL and the reference method remained statistically significant with systematic bias towards overestimation	https://pubmed.ncbi.nlm.nih.gov/28389913/
Rubenstein et al (2018)	RE-CS	EBL vs. Triton System in terms of clinical outcome of patients	Triton (756)<br>CG (2025)	-the device identified bleeding more frequently than EBL<br>-similar transfusion rates in both groups, in the Triton group fewer RBC were administered and none of the patients received plasma or cryoprecipitate<br>-in the group with EBL these products were administered	https://pubmed.ncbi.nlm.nih.gov/29207419/
Fedouruk et al (2019)	P-OS	Triton system vs. gEBL vs. EBL in relation to the prediction of the postoperative HB value	SG (61)	-statistically significant, but weak correlation was observed between the Triton system and the Hb value 10 minutes after arrival at the post-anaesthesia unit (PACU) (r = -0.33)<br>-no statistically significant correlations between the other methods and the PACU Hb value	https://pubmed.ncbi.nlm.nih.gov/29847380/
Saoud et al (2019)	P-CS	EBL vs. Triton system	SG (242)	-Triton device estimated the mean blood loss significantly lower than EBL<br>-Triton system correlated better with the HB difference between pre and postoperative	https://pubmed.ncbi.nlm.nih.gov/31229429/
**Miscellaneous Methods**					
Torella et al (2002)	P-OS	Near infrared spectroscopy (NIRS) to determine blood loss during a voluntary blood donation	SG (10)	-good correlation between NIRS parameters and blood loss	https://pubmed.ncbi.nlm.nih.gov/12062210/
Torella et al (2002)	P-OS	NIRS to determine blood loss during a voluntary blood donation	SG (40)	-regional tissue oxygenation decreases proportionally to the uncompensated blood loss	https://pubmed.ncbi.nlm.nih.gov/12412623/
Lyon et al (2005)	P-OS	Measurement of the change of IVCd in relation to blood loss in voluntary blood donations	SG (31)	-change was approximately 5 mm and was consistent regardless of initial diameter	https://pubmed.ncbi.nlm.nih.gov/15672337/
Resnick et al (2011)	P-OS	Usefulness of ultrasound to detect signs of haemorrhagic shock in volunteer blood donors compared to changes in vital signs	SG (38)	-serial changes in the IVCd after a blood donation of 500ml is not clinically significant	https://pubmed.ncbi.nlm.nih.gov/21421294/
Oba et al (2019)	P-CS	Usefulness of ultrasound to detect signs of PPH	IG (7)<br>CG (77)	-the ultrasound measurement of the IVCd compared to the vital parameters is a more accurate predictor of severe anaemia after transvaginal delivery	https://pubmed.ncbi.nlm.nih.gov/29621917/
Oshima et al (2005)	P-CS	Continuous non-invasive intraoperative HB monitoring using HemoCue to determine blood loss vs. EBL	SG (23)	-no significant correlation between the CBL and the difference in Hb level was found	https://pubmed.ncbi.nlm.nih.gov/16113493/
Meunier et al (2008)	P-CS	Continuous non-invasive HB monitoring to determine the lost blood volume in voluntary blood donations	group 1 (21)<br>group 2 (18)	-blood loss was underestimated by more than 60% compared to the ABL ($P < 0.00001$)	https://pubmed.ncbi.nlm.nih.gov/18510580/
Kamal et al (2016)	DB-R-C-CT	Continuous non-invasive Hb monitoring to determine the lost blood volume	laboratory (50)<br>Masimo (50)	-the number of transfused red blood cell units (RBC) was significantly lower in the HB monitoring group than in the CG (p value 0.02)<br>-the delay time before transfusion was statistically significantly lower in the HB monitoring group (10.5 ± 2.3) than in the CG (43.5 ± 8.6)	https://www.scirp.org/journal/paperinformation.aspx?paperid=64633

Mannova et al (2013)	Matched-CS	Haemodynamic monitoring by oesophageal Doppler	Doppler (70)<br>CG (70)	-patients monitored with Doppler received more fluid perioperatively, had on average shorter stays in intensive care units and shorter overall stays compared to the control group	https://pubmed.ncbi.nlm.nih.gov/23331203/
Imai et al (2017)	P-CS	contrast-enhanced ultrasound (CEUS) in detecting PPH	SG (37)	-there were no false positive CEUS findings<br>-CEUS was positive in 6 of 37 patients and 7 of 37 patients had PPH	https://pubmed.ncbi.nlm.nih.gov/28024660/
				systematic review (SR); prospective (P); single-blinded (SB); double-blinded (DB); study (S); observational study (OS); clinical trial (CT); cross sectional study (CSS); randomized (R); cohort study (CS); controlled (C); retrospective chart analysis (RCA); comparative study (COS); retrospective (RE); in vitro validation study (VVS); open (O); crossover study (CRS); descriptive study (DS); study group (SG); controll group (CG); intervention group (IG); direct measurement (DM); visual estimation (EBL); calculated blood loss (CBL); gravimetric method (gEBL); red blood cell units (RBC); haemoglobin (HB); haematocrit (HCT); acid hematin method (AHM); influencing factors (IF); actual blood loss (ABL); combination of direct measurement and gravimetric method (MBL); intra-class correlation (ICC)	
