# Supplementary material for: Comparison of common perioperative blood loss estimation techniques: a systematic review and meta-analysis
Source: J Clin Monit Comput. 2020 Aug 19;35(2):245–58. doi: 10.1007/s10877-020-00579-8 (PMC7943515; doi:10.1007/s10877-020-00579-8)
Supplement: Supplementary file 2 — Supplementary file2 (DOCX 103 kb) [file 10877_2020_579_MOESM2_ESM.docx]

***Supplement 2***

*Overview of studies investigating visual estimation of blood loss*

The visual estimation of blood loss by clinicians is not only one of the most widely used methods, but also the most examined one. This includes the estimation of blood volumes in sponges and suction containers but also the recording of external blood losses. Forty-eight studies dealt with the accuracy and improvement of visual assessment and 29 of these were performed in obstetrics.

Nine studies [30–38] including overall 655 participants examined whether the variables gender, age and work experience influence accuracy and did not identify any significant impact. In contrast, two observational studies [40, 41] including overall 212 participants showed that clinicians with less experience tend to estimate more accurately than clinicians with more experience. McCullough et al [42] could not find a significant difference in a retrospective chart analysis of 52 cases between the estimates of surgeons and anaesthetists.

Conversely, four studies [38, 43–45] including 420 participants showed that anaesthetists estimate more accurately. However, almost all studies concluded a significant inaccuracy of the visual estimation compared to the respective reference method. Kavle et al [46] concluded that the visual estimation of midwives (n=158) is sufficiently accurate compared to the alkaline haematin technique. Howe et al [47] predicted postoperative Hb-level using the visually estimated blood loss. The retrospective chart analysis of the estimated blood loss versus the Hb difference showed a very low correlation in 198 cases.

The resulting question about general tendencies of over- and underestimation is controversially answered in literature. For example, the investigations of four studies [29, 35, 41, 48] including 615 participants showed a tendency to overestimate whereas 12 studies [7, 8, 31, 41, 43, 45, 49–54] including 7,744 participants showed a tendency to underestimate.

The hypothesis that with increasing blood loss, the estimation becomes less accurate is supported by the investigations of three studies [7–9] including 726 participants. Buckland et al [6] and Mbachu et al [5] observed in their simulated clinical scenarios, including 232 participants, that blood in containers is better rated than blood on objects and floors. Maslovitz et al [55] found an improvement of accuracy of visual estimation for total blood loss through simulation-based practical training when the intermediate blood loss is estimated at regular intervals and then summed up (24 participants out of 126). Several studies [5, 7, 21, 33, 36, 38, 56] including 941 participants also found an improvement in visual estimation through live-based and web-based training in simulated scenarios. For example, Sukprasert et al [56] performed a randomized controlled clinical trial involving 90 participants to test the improvement of visual estimation through didactic sessions and found a significant improvement after the training. Similarly, Toledo et al [21] compared the effectiveness of live training with that of web-based training (2010) and evaluated the knowledge of the participants (web-based training) 9 months later [57].

Mean aggregated pre-test accuracy of blood loss estimate was -38% (-59% to -20%) and post-test accuracy was -4% (-7% to 13%). There was no difference in improvement between live and web-based training. In the follow-up, the participants completed the same post-test stations as after the first training using photos. The median aggregated pre-test accuracy of the blood loss estimate of the web-based training participants was 47.8% (60.9% to 28.7%) with an improvement to 13.5% (18% to 9.8%) in the immediate post-test. At the 9-month follow-up the accuracy had decreased to 34.6% (53.4% to 14.3%). An improvement in the accuracy of visual estimation by visual aids was noted by seven studies [32, 58–63] including 417 participants. In contrast, Nelson et al [64] found an overestimation through the use of a pictogram (n=81). Homcha et al [32] hypothesized that imaging of materials with different blood volumes and amniotic fluids commonly used during caesarean section would improve clinician accuracy in visual blood loss estimates. Indeed, there was an improvement from 41% accuracy without visual aids to 74% with aids averaged over the 46 participants. In contrast, Brooks et al [65] observed that in all cases (n=69) the estimates of the participants were significantly more accurate when only the collection bag with the baby scale was used without visual aids.
