# Supplementary material for: Comparison of common perioperative blood loss estimation techniques: a systematic review and meta-analysis
Source: J Clin Monit Comput. 2020 Aug 19;35(2):245–58. doi: 10.1007/s10877-020-00579-8 (PMC7943515; doi:10.1007/s10877-020-00579-8)
Supplement: Supplementary file 3 — Supplementary file3 (DOCX 38 kb) [file 10877_2020_579_MOESM3_ESM.docx]

**Supplement 3**

*Overview of studies investigating gravimetric estimation of blood loss*

The gravimetric method is an indirect measurement of blood loss. Blood loss can be deduced by weighing the surgical material contaminated with blood and subtracting the dry weights. By summing up the measured weight of the blood and estimating the fluid in the suction container, the blood loss can be calculated with a conversion of 1g=1ml blood [38, 66, 69].

Three studies [27, 67, 68] compared the gravimetric method with the spectrophotometer and all observed a significant positive linear correlation. However, there was a systemic bias in favour of overestimating blood loss. Doctorvaladan et al [70] observed an overestimation of blood volume by the gravimetric method in 34% of cases (n=17).

Lilley et al [29] found that the correlation between the gravimetric method and the corrected Hb drop was higher for blood loss over 1500ml.

Atukunda et al [69] evaluated weighing as a cost-effective and valid method for diagnosing postpartum haemorrhage (PPH) in low-resource countries. According to the gravimetric method, 23% of the 1,140 women had a blood loss above 500ml (Definition of the author of PPH). If PPH is defined as a Hb decrease greater than 10% from baseline, 22.6% of the women had PPH. Fedoruk et al [39] did not observe a significant correlation between the blood loss determined by weighing and the Hb values measured 10 minutes after arrival at the post-anaesthesia ward. A survey by Ladouceur et al [12] found that 69% (n = 18) of the participants thought that QBL (Quantification of Blood Loss: a combination of weighing, direct measurement and visual estimation) was the best approach. When asked whether the calibrated drapes and scales are easy to handle, 54% (n = 13) agreed, 25% (n = 6) disagreed.
