# Supplementary material for: Comparison of common perioperative blood loss estimation techniques: a systematic review and meta-analysis
Source: J Clin Monit Comput. 2020 Aug 19;35(2):245–58. doi: 10.1007/s10877-020-00579-8 (PMC7943515; doi:10.1007/s10877-020-00579-8)
Supplement: Supplementary file 4 — Supplementary file4 (DOCX 30 kb) [file 10877_2020_579_MOESM4_ESM.docx]

**Supplement 4**

*Overview of studies investigating direct measurement of blood loss*

The direct measurement of blood loss is a simple and long-established method that is mainly used in the field of obstetrics. Nine studies focused on calibrated collection bags specially designed for vaginal deliveries. These are available from various providers and can be purchased for about one USD. The collector bag is placed under the woman's buttocks immediately after the birth of the child and collects all fluids. At the bottom of the plastic foil there is a calibrated collector bag with a scale on which the current blood loss can be read.

Patel et al [71] compared the direct measurement with photo spectroscopy in a randomized controlled clinical study with 62 participants and concluded that the collection bag has a high sensitivity and specificity for the diagnosis of PPH. Zhang et al [72], on the other hand, found no significant difference in the number of PPH diagnosed in their comparison between visual estimation (n=14,244) and direct measurement (n= 11,037) in a cluster-randomized clinical trial. Tixier et al [73] found in their cohort study with 122 participants that the probability that the bag contains 500ml when a patient has a true PPH is 66.7% with a specificity of 94.2%. Legendre et al [74] investigated the accuracy of estimating blood loss in obstetricians with the collection bag. In total, 96 of 98 participants correctly estimated blood loss for smaller volumes. For larger volumes, the accuracy of the participants was slightly reduced.

It is also possible to use cholera beds to directly measure blood loss during vaginal delivery. Through an opening at buttock level, the blood can flow directly into a container underneath the bed and be collected there. According to Strand et al [25], this method is easy to use and improves the visual estimation of blood loss, especially in resource-poor areas.
