# Supplementary material for: Comparison of common perioperative blood loss estimation techniques: a systematic review and meta-analysis
Source: J Clin Monit Comput. 2020 Aug 19;35(2):245–58. doi: 10.1007/s10877-020-00579-8 (PMC7943515; doi:10.1007/s10877-020-00579-8)
Supplement: Supplementary file 5 — Supplementary file5 (DOCX 51 kb) [file 10877_2020_579_MOESM5_ESM.docx]

**Supplement 5**

*Overview of studies investigating calculation of blood loss*

Currently, various mathematical approaches are used in clinical practice to evaluate blood loss. In order to calculate the most exact intraoperative blood loss, the formula has been modified over time. Thus, three different formulas are used in the literature to calculate the total blood volume of patients. The Moore formula [75, 76], which, like Nadler's formula [75–81] takes into account height, weight and sex for the calculation, and the ICSH formula [75, 76], which uses gender and body surface area to calculate the total blood volume. The formula according to Nadler was used most often in literature. In ten studies analysed, 11 different formulas for calculating intraoperative blood loss were used.

The formula according to Wards [76], Flordal [78, 79] and Brecher [81] uses a natural logarithm function to calculate blood loss. Brecher [81] and Flordal [78, 79] divide the preoperative Hb-level by the postoperative Hb-level and Wards does the same with the pre- and postoperative haematocrit values (Hct). Two studies [78, 79] used the formula according to Flordal and came to different results. Božičković et al [78] observed no significant correlation between collected and calculated blood volumes for 14 participants because transfusions were not included in the calculation. Milosevic et al [79] excluded women with blood transfusions and observed a statistically significant correlation between collected and calculated blood loss for 1,487 participants. The Bourke and Smith formula [75, 76] is based on the Wards formula, but the natural logarithm was replaced by three minus the mean perioperative Hct.

In the Gross formula [75, 76, 82], the total blood volume is multiplied by the difference between pre- and postoperative Hct. The Mercuriali [75] and Camarasa formulas [75] also use the difference between pre- and postoperative Hct, but transfusions are also taken into account and there is a differentiation between autologous and homologous transfusions and those from blood recovery systems. Lopez-Picardo et al [75] compared the Bourke and Smith formula and the Gross formula with the Camarasa formula (this one for reference) for 105 participants. A good correlation of both formulas with the reference was found. It was observed that when the volume of blood loss was low, the level of agreement between the formulae was high.

The Lopez-Picardo formula [76] is an evolution of the Camarasa formula and uses the ICSH (International Council for Standardization in Haematology) formula instead of the Moore formula to calculate the total blood volume. The OSTHEO formula (Orthopedic Surgery Transfusion Haemoglobin European Overview) [76, 82] also uses the ICSH formula to calculate the total blood volume. The total blood volume is then multiplied by pre- and postoperative Hct. The uncompensated erythrocytes loss is then calculated from the haematocrit values and added to the transfused red blood cell units (RBC) volume.

The Hb difference method [76, 82] distinguishes between allogeneic and autologous transfusions and uses the difference between pre- and postoperative Hb-level for blood loss calculation. Jaramillo et al [76] compared the Lopez-Picardo formula, the OSTHEO formula, the Hb difference method, the Wards formula, the Bourke and Smith formula and the Gross formula with direct measurement as reference. Among the formulas, the López-Picado formula reached the concordance correlation coefﬁcient index at each postoperative time. All blood loss estimation formulas showed a significant tendency to overestimate blood loss.
