# Supplementary material for: Comparison of common perioperative blood loss estimation techniques: a systematic review and meta-analysis
Source: J Clin Monit Comput. 2020 Aug 19;35(2):245–58. doi: 10.1007/s10877-020-00579-8 (PMC7943515; doi:10.1007/s10877-020-00579-8)
Supplement: Supplementary file 6 — Supplementary file6 (DOCX 40 kb) [file 10877_2020_579_MOESM6_ESM.docx]

**Supplement 6**

*Overview of studies investigating colorimetric estimation of blood loss*

A smartphone application (Triton^TM^) developed by Gauss Surgical Inc. is able to calculate blood loss by taking photographs of used surgical gauze and canisters. The colorimetric technique analyses photographic and geometric information from relevant areas, with the aim of automatically filtering out the effects of non-blood components mixed in each sponge and canister and calculating the Hb mass present in the gauze or canister from the image. By entering the preoperative Hb-level, the blood loss can then be calculated. In the studies analyzed, high degrees of correlation with the reference blood.

A total of ten studies [22, 24, 26–28, 39, 67, 68, 70, 83] were analysed, that used the Triton system. To verify the accuracy of the system, Konig et al [26] performed an in vitro study and used the Hb-assay as a reference method. They observed a strong positive correlation between the Triton system and the Hb assay for all surgical drapes over the range of intraoperative environmental conditions tested. However, there were significant deviations in Hb measurements with respect to the tissue mark. The Triton system tended to underestimate Hb loss in surgical drapes with higher Hb content and in dark ambient light settings.

Sharareh et al [27] observed in 50 cases a significant positive correlation and a mean deviation of 6.4g Hb between Triton system and assay values. Konig et al [28] also validated the system extension for canisters. Different Hb concentrations, volumes and haemolysis degrees were simulated under different light conditions and tested to what extent drying out changes the measurement results. The measurements of the Triton system correlated well with the Hb assay. Scanning the dried surgical drapes two hours after surgery showed that drying did not significantly change the results.

Thurer et al [22] investigated the usefulness of the system and compared it with the visual estimation. Here the actual postoperative Hb-level served as a reference and the values of the visual estimation and the Triton system were used in the same formula to predict the postoperative Hb-level. The Hb-levels in the Triton group (n=167) were a more sensitive predictor of the actual Hb-level on the first postoperative day than the calculated values in the historical group (n=100). Rubenstein et al observed a stronger correlation of Hb-levels on the first postoperative day with the actual value in patients in the device group (n=756) with an estimated blood loss of more than 1,000 ml than the predictions using the visually estimated blood loss for patients (n=2,025) whose visual estimate was more than 1000 ml. Fedoruk et al compared the Hb value measured by Triton with the Hb-level measured ten minutes after arrival at the post-anaesthesia unit (PACU) and found a statistically significant but relatively weak correlation.
