# Supplementary material for: Comparison of common perioperative blood loss estimation techniques: a systematic review and meta-analysis
Source: J Clin Monit Comput. 2020 Aug 19;35(2):245–58. doi: 10.1007/s10877-020-00579-8 (PMC7943515; doi:10.1007/s10877-020-00579-8)
Supplement: Supplementary file 7 — Supplementary file7 (DOCX 38 kb) [file 10877_2020_579_MOESM7_ESM.docx]

**Supplement 7**

*Overview of miscellaneous methods for estimation of blood loss*

Other methods to record blood loss intraoperatively are only described in ten studies. According to Lyon et al [84] and Oba et al [20], ultrasound of the inferior vena cava can be used to estimate blood loss. Resnick et al [85] concluded that serial changes in the diameter of the vena cava inferior after a blood donation of 500ml are not clinically significant.

Imai et al [86] used contrast-enhanced ultrasound (CEUS) to detect PPH. The contrast medium enters the uterus during bleeding and becomes visible by ultrasound. The incidence of excessive bleeding is 18.9% (7 out of 37 patients). CEUS was positive in six patients and remained positively associated with an increased probability of excessive bleeding. There were no false positive CEUS findings.

Mannova [19] examined whether hemodynamic Doppler monitoring of patients shortens the length of hospital stay and mortality. The oesophageal Doppler technique measures the blood flow velocity in the descending aorta using the Doppler transducer.

When comparing the Doppler group (n=70) with the control group (n=70), the former showed shorter median stay in intensive care and a shorter Length of stay. Comparing the frequency of postoperative complications, a statistically significant difference in the frequency of serious complications was found.

Torella et al [87] used near-infrared spectroscopy to detect blood loss. Near-infrared spectroscopy measures regional Hb saturation from deeper tissues, assuming that the ideal transfusion trigger should be able to identify the moment when the oxygen supply in the tissue is no longer sufficient in relation to the tissue metabolism.

Torella's [90] study showed that cSO2 (Hb saturation of left frontal lobe), pSO2 (Hb saturation of left calf) and HbD (oxygenation index) measured by near-infrared spectroscopy decreases proportionally to the uncompensated blood loss. Then the study was extended from ten to 40 participants and it was found that regional oxygen supply also decreased proportionally to blood loss in subjects whose blood loss was insufficient to alter vital functions.

In three other studies [24, 88, 89] the use of continuous non-invasive intraoperative Hb-monitoring to determine blood loss intraoperatively was examined.

Meunier et al [23] compared the continuous Hb-monitoring with the Hb-level on day one after blood donation in 39 participants and found that the blood loss was underestimated by more than 60%.

Kamal et al [89] evaluated continuous non-invasive Hb measurement as a guide for blood transfusions. The number of transfused RBC units was significantly lower in the study group (n=50) than in the control group (n=50). The delay time before transfusion was statistically significantly lower in the study group than in the control group.
